# Fortification of pork loins with docosahexaenoic acid (DHA) and its effect on flavour

**DOI:** 10.1186/2049-1891-4-46

**Published:** 2013-11-20

**Authors:** William J Meadus, Tyler D Turner, Michael ER Dugan, Jennifer L Aalhus, Pascale Duff, David Rolland, Bethany Uttaro, Lorna L Gibson

**Affiliations:** 1 Agriculture and Agri-Food Canada, Lacombe Research Centre, 6000 C&E Trail, Lacombe T4L 1 W1AB, Canada

**Keywords:** Docosahexaenoic acid, Injection marinade, Pork, Sensory characteristics

## Abstract

Pork is traditionally low in docosahexanoic acid (DHA, C22:6n-3) and deficient in omega-3 fats for a balanced human diet. DHA as triglycerides was commercially prepared from the microalgae *Schizochytrium* and injected into fresh pork loins. Treatments of a mixed brine control (CON), 3.1% sunflower oil in mixed brine (SF) and a 3.1% DHA oil in mixed brine (DHA) were injected into pork loins at 10 mL/100 g and grilled at 205°C. After cooking, the CON and SF pork loins contained 0.03 to 0.05 mg DHA/g of pork and the DHA injected loins contained approximately 1.46 mg DHA/g. This also changed the fatty acid profile of omega-6: omega-3 from, 5 to 1 in the CON pork, to a ratio of 1.7 to 1 in DHA pork. The appearance, odor, oxidation rates and sensory taste, as judged by a trained panel, determined the DHA injected meat to be, 'slightly desirable’ and gave lower 'off flavour’ scores, relative to the CON and SF injected pork. Pork can be fortified with DHA oil to 146 mg/100 g serving, which would meet half the recommended daily omega 3 fatty acid requirements for adult humans and would be desirable in taste.

## Background

Pork is viewed as a lean healthy food, providing good nutrition; however, there are concerns about the quantity and types of fat it possesses. According to the USDA, a typical pork chop contains 11.3 g of fat/100 g of meat, of which 1.3 g is polyunsaturated fat and essentially no omega-3 fats [1]. Humans require the essential fatty acids omega-6 linoleic acid (C18:2n-6) and omega-3 α-linolenic acid (C18:3n-3) in their diet. Human adults are recommended to consume at least 1 g/d of omega-3 fat for proper cardiovascular health [2,3]. The long chain omega-3 fatty acid, docosahexaenoic acid (C22:6n-3), is particularly important, since it comprises ~14% of the cerebral cortex [4,5]. To improve the omega-3 nutritional content of pork, researchers have fed plants such as, flax [6], soybeans and canola [7] which are high in α-linolenic acid; however, α-linolenic acid is only weakly converted to DHA [5]. Pork can be selectively enriched with DHA by feeding fish oils such as tuna [8] or by feeding microalgae biomass *Schizochytricium*[9]. However, there are problems with 'off’ flavours and trimethylamine odors caused by fish sources [8,10,11] or with achieving adequate concentrations of expensive pure sources of dietary grade DHA. The option of directly injecting the DHA into the meat as a brine marinade, may overcome some of these issues.

Injecting water for moisture into pork has been in practice since 1960 [12]. The addition of a polyphosphate to a brine mixture further improves the juiciness, tenderness and flavour after cooking [13]; however, some discoloration has been noted. In addition to brine, injection of fats and oils [14] may improve the eating experience of pork. In North America, lean pork loins are averaging less than 2% intramuscular fat (IMF), the minimal IMF for consumer acceptance is > 3% [15]. The IMF adds flavour and juiciness and has a minor improvement on tenderness [16]. Beef injections with conjugated linoleic acid has recently been done to improve the nutrition but also to improve the eating quality experience of beef [17]. This study was done to improve the nutritional profile of pork by injecting lean pork loins with DHA oil and to assess consumer perceptions of eating quality and to examine if any off flavours would be generated by the DHA oil.

## Methods

### Chemicals

Docosahexaenoic acid oil was supplied by Martek Bioscience Corporation (Boulder, CO, USA). Sunflower oil 100% was purchased from Compliments Company (Mississauga, ON, Canada). Sodium tripolyphoshate and salt was supplied by the Food Supplies Company (Winnipeg, Manitoba, Canada). The soy lecithin was from Solae, St. Louis, MO USA. Alpha tocopherol acetate was from Aquas Chem. Intl. (Torrance, CA, USA). Thiobarbituric acid, propyl gallate, ethylenediaminetetraacetic acid (EDTA), malonaldehyde, tetraethoxypropane, 1-hexanal, butanoic acid were purchased from Sigma-Aldrich Canada (Oakville, ON, Canada).

### Animals

Animals used in this study were cared for and slaughtered, according to Canadian Council for Animal Care guidelines [18]. Barrows were selected from the Lacombe Research Centre f1 pig herd produced from Large White X Duroc mating. Pigs were given water *ad libitum* and fed a standard finisher diet comprised of 35% corn, 25% peas, 19% barley, 17% canola and 4% vitamin premix including 100 IU/kg of α-tocopherol (vitamin E) and 0.5 mg/kg selenium [19]. The animals (n = 20) were slaughtered at the Lacombe Research Centre abattoir at 120 kg after a 24 h feed withdraw but with full access to water. Carcasses were split and cooled for 24 h at 4°C, then 24 carcass halves were selected and cut into primals, according to Canadian Meat Council guideline [20]. The ~10 kg boneless loins were removed from both sides of the carcass, weighed, and distributed for treatment. Loins were evaluated and judge equal, based on visual colour and marbling scores [20].

### Injection treatment of pork loins

Three treatments were allocated to the 24 h boneless loins (n = 8 loins/treatment). The treatments were an injection of 10 mL/100 g loin (longissimus dorsi muscle) of mixed brine solution (CON) containing phosphate, sodium chloride, a 3.1% sunflower oil in the mixed brine solution (SF) or a 3.1% DHA oil in mixed brine solution (DHA). The mixed brine consisted of, 4.8% sodium tripolyphosphate Na_5_P_3_O_10_ (BCCHEM, PQ, Canada), 4.8% sodium chloride, 0.01% α-tocopherol, and 0.15% precept 8140 powdered soy lecithin in distilled water. The SF oil consisted of the control brine mixed with 3.1% of mid oleic grade sunflower oil (Compliments, ON, Canada). The DHA oil consisted of the control brine mixed with 3.1% of DHA-S oil (Martek Bioscience Corp, Boulder, CO, USA). DHA-S oil was comprised of 35% docosahexaenoic acid extracted from microalgae mixed with 65% high oleic sunflower oil, 0.02% α- tocopherol and 0.01% soy lecithin.

The injection of pork loins with 3.1% DHA or 3.1% sunflower oil in a tripolyphosphate brine solution would add approximately 0.31 mL of oil/100 g of pork. The brine mixtures were injected using 4 mm needles spaced 2.8 cm in an Inject Star BI-72 unit (J Redmond & Sons, Northampton, UK), set at 2 bar and 56 strokes/min. After injection, the loins were allowed to equilibrate for 18 h at 2°C and then cut into 1 inch chops from the center, yielding 8–10 chops/loin, and 8 loins/treatment. The fluid loss was not measured at cutting. The chops from the three treatment groups, were packaged individually in polystyrene trays on dri-loc pads (UZ Soaker Ultra Zap Pads, Paper Pak Industries Washington, GA, USA), overwrapped with oxygen permeable film (8,000 mL/m^2^/24 h vitafilm choice wrap (Goodyear Canada Inc, Toronto, ON, Canada) and stored for an additional 24 h at +4°C. Day 1 raw chops (approximately 66 h *post mortem*) were selected (2/loin/treatment) for evaluation by trained panellists for visual colour, striping caused by the injections and odours and measured for color and thiobarbituric acid (TBARS) and again after 3 d, under refrigeration at 2°C, the maximum reasonable limit for retail display [21,22]. A portion of the chops from each of the three treatments were sealed (n = 12/treatment) immediately after cutting, in vacuum packages (Multivac AGW; Multivac Inc., Kansas City, MO, USA) and stored in a -20°C freezer for the FAME analysis.

### Fatty acid analysis (FAME) of oil and raw pork loins

Fatty acid methyl esters (FAME) extracts were isolated from the DHA and SF oils (Table 1). FAME extracts were also isolated (4/treatment) from CON, SF and DHA thawed raw chops and then the same chops were cooked at 205°C to an internal temperature of 71°C and then resampled. The samples were prepared according to the one-step extraction trans-esterification procedure [23]. Thawed raw and cooked pork loins (1 g) were pureed by blending with a Blixer 3, RoboCoupe (Ridgeland, MS,USA) food processor in 10 mL of 2:1 chloroform/methanol and passing a through a 70 μmol/L glass filter. The FAME were extracted from the filtrate in 3 mL of hexane and then dried over sodium sulfate. Extracted lipids were methylated according to [24]. FAME was recovered in hexane prior to gas chromatography injection. FAME were analyzed using a Varian 3800GC (Varian, Walnut Creek, CA, USA) equipped with a Varian 8400 auto-sampler and a 30 m SP2340 capillary column (Supelco, Bellefonte, PA) with flame ionization detection. The injector and FID detector were set 250°C and gas flow at a constant 15 psi. Chromatograms were integrated using Varian Star Chromatography Workstation software. Peaks were identified using a GC reference standard GLC463 from Nu-Check-Prep, Elysian, MN, USA). The iodine value of the fatty acids was calculated by multiplying the percentage of each fatty acid (Tables 1 &2) contained in the sample by the Iodine number of the fatty acids [25].

**Table 1:** Fatty acid methyl ester profile of the DHA oil and Sunflower oil preparations

**FAME, (mg/g)**	**DHA oil, (mg/g)**	**Sunflower oil, (mg/g)**
C14:0	68.41	0.40
C16:0	166.37	39.66
C16:1cis9	2.62	0.61
C18:0	7.08	33.00
C18:1cis9	99.76	565.21
C18:1cis11	1.60	4.41
C18:2n-6	8.49	273.12
C18:3n-6	2.39	2.55
C18:3n-3	0.55	1.63
C20:1cis11	0.27	1.96
C20:2n-6	1.81	8.40
C20:3n-6	3.12	0.00
C20:4n-6	2.99	0.00
C20:5n-3	8.47	0.00
C22:5n-6	146.20	0.00
C22:5n-3	4.75	0.00
C22:6n-3	394.15	0.00
Total FAME mg/g	949.50	935.85
Iodine Index^1^	271.36	110.43

^1^Estimated iodine index calculated by the % of fatty acid with the sample multiplied by the iodine value of the fatty acid.

**Table 2:** Fatty acid methyl esters profile of the raw and cooked injected pork loins between Control brine (CON), Sunflower (SF) and DHA treatments

**FAME**	**Raw**			**Cooked**				***P *** **value**	
**mg/g wet tissue**	**CON**	**SF**	**DHA**	**CON**	**SF**	**DHA**	***SEM***	**Treatment**	**Cooking**
C14:0	0.62^a^	1.24^b^	1.27^b^	0.55^a^	1.44^c^	1.68^c^	*0.130*	0.005	0.004
C16:0	10.90^a^	22.28^c^	18.08^b^	10.54^a^	26.65^c^	24.64^c^	*1.524*	0.001	0.001
C16:1cis9	1.16^a^	2.12^b^	1.93^b^	1.11^a^	2.60^c^	2.70^c^	*0.164*	0.002	0.002
C18:0	5.68^a^	12.26^c^	8.87^b^	5.74^a^	14.78^d^	12.09^c^	*0.833*	0.001	0.001
C18:1cis9	15.96^a^	32.87^c^	24.59^b^	15.29^a^	40.42^c^	33.79^c^	*2.002*	0.001	0.001
C18:1cis11	2.42^a^	5.28^b^	3.92^a^	2.13 ^a^	5.61^b^	4.95^b^	*0.335*	0.001	0.177
C18:2n-6	3.05^a^	5.47^b^	4.26^b^	3.05^a^	6.64^c^	5.14^b^	*0.414*	0.006	0.001
C18:3n-3	0.40^a^	0.77^b^	0.69^b^	0.32^a^	0.87^b^	0.76^b^	*0.089*	0.022	0.296
C18:3n-6	0.09^a^	0.23^b^	0.16^b^	0.09^a^	0.28^b^	0.20^b^	*0.021*	0.003	0.005
C20:1cis11	0.41^a^	0.96^b^	0.62^b^	0.37^a^	1.16^c^	0.83^b^	*0.075*	0.002	0.004
C20:2n-6	0.08^a^	0.16^c^	0.12^b^	0.07^a^	0.21^d^	0.15^c^	*0.016*	0.007	0.023
C20:3n-6	0.06	0.08	0.07	0.05	0.10	0.10	*0.009*	0.018	0.258
C20:3n-3	0.03	0.06	0.05	0.03	0.07	0.06	*0.008*	0.062	0.102
C20:4n-6	0.28^a^	0.34^b^	0.31^b^	0.33^b^	0.45^c^	0.37^b^	*0.022*	0.057	0.001
C20:5n-3	0.04	0.04	0.07	0.06	0.07	0.08	*0.005*	0.016	0.002
C22:0	0.01	0.03	0.02	0.01	0.02	0.02	*0.003*	0.009	0.073
C22:5n-6	0.00^a^	0.00^a^	0.44^b^	0.01^a^	0.00^a^	0.54^b^	*0.025*	0.001	0.108
C22:5n-3	0.10	0.15	0.13	0.12	0.21	0.18	*0.016*	0.037	0.002
C22:6n-3	0.02^a^	0.04^a^	1.16^b^	0.03^a^	0.05^a^	1.46^b^	*0.068*	0.000	0.075
SFA^1^	17.30^a^	36.05^c^	28.41^b^	16.93^a^	43.17^c^	38.63^c^	*2.510*	0.001	0.001
MUFA^2^	19.95^a^	41.23^c^	31.06^c^	18.90^a^	49.78^c^	42.28^c^	*2.576*	0.001	0.001
PUFA^3^	4.07^a^	7.10^b^	7.30^b^	4.06^a^	8.67^c^	8.83^c^	*0.673*	0.004	0.000
Total FAME	41.32^a^	84.38^c^	66.76^b^	39.88^a^	101.62^c^	89.73^c^	*1.920*	0.002	0.001
Iodine Index^4^	27.15^a^	53.09^b^	46.65^b^	26.32^a^	64.48^c^	60.95^c^	*3.184*	0.001	0.001

^abcd^ Means within rows with unique superscript, differ significantly (P > 0.05). SEM; standard error of means, within row.

^1^SFA; saturated fatty acids, with no double bonds.

^2^MUFA; monounsaturated fatty acids, with one double bond.

^3^PUFA; polyunsaturated fatty acids, with two or more double bonds.

^4^Estimated iodine index calculated by the% of fatty acid with the sample multiplied by the iodine value of the fatty acid.

### Colour measurements

The colour of each loin treatment section was measured using a Minolta CM2002 color meter (Minolta Canada Inc., ON, Canada). Chops were cut from the injected treated loin and allowed to oxygenate at 4°C for 20 min before taking the colour measurements directly from the meat surface. The CIE L*, a*, b* colour coordinates were recorded along with Chroma and hue values and illuminated using a Minolta CR-300 color meter on the raw injected chops at days 0, 1 and 3 according to the manufacturers specification (Konica Minolta, Ramsay, NJ, USA).

### Thiobarbituric Acid Reactive Substances (TBARS)

The free meat juice purge (1 mL) was collected from the drip trays (n =8/treatment) of the raw 1d and 3d, injected loin chops and then the chops were diced into 1 g cubes and blended with an Ultra Turax in 10 mL of extraction solution: trichloroacetic acid (75 g of TCA/L in water), propyl gallate (1 g/L) and EDTA (1 g/L). The extraction solution was filtered through a Whatman no. 42 filter then 2.5 mL of the filtered extract was mixed with 2.5 mL of thiobarbituric acid (TBA) (2.88 g/L) and heated to 94°C for 40 min. in closed glass vials. The samples were immediately cooled, and the absorbance was measured at 531 nm. TBARS values were determined relative to a standard curve of malonaldehyde generated with 1 g/L of tetraethoxypropane and 20 mmol/L to 90 mM TBA solution [26].

### Sensory and odours evaluation of raw loin chops

Panellist (*n* = 8) were informed, selected and trained, according to the American Meat Science Associations guidelines [27]. The panellist were asked evaluate the visual display of the 0 d and 3 d raw loin chops and give rating based on a 8-point hedonic scale for: overall retail appearance (8 = extremely desirable to 1 = extremely undesirable) and descriptive scales for lean muscle color (1 = pale pink/grey and white to 6 = dark purplish red), colour of striping (1 = none to 7 = yellow/brown),% striping (1 = none to 7 = 100%), spoilage colour (1 = none to 7 = brown),% surface spoilage (1 = none to 7 = 100%), and visual marbling score (1 = devoid to 6 = abundant).

Odour rating was completed using a 4-point descriptive scale for Off odor intensity (4 = prevalent to 1 = no off odours) a 5-point hedonic scale for odour acceptability (5 = unacceptable to 1 = acceptable), and a 9-point descriptive classification for Off odours (9 = other, 8 = unidentified, 7 = fishy, 6 = rancid/painty, 5 = stale/cardboard, 4 = piggy/barn like, 3 = metallic, 2 = off/sour, 1 = none). The panellist were also asked to rate the 3 brine mixtures.

### Sensory and odours evaluation of cooked loin chops

Assessment of cooked chops was performed on 1 d loins, 24 h after brine injection and approximately 66 h *post mortem*. Each treated loin was weighed after removal from the vacuum pack and the percentage cooking loss was calculated based on the weight, before and after cooking. The injected loin chops, 8/treatment, were sliced into 1 inch chops and then cooked on a preheated Garland electric grill ED-30B at 205°C. The chops internal temperature was monitored every 5 s with a type T thermo-coupled temperature probes until the internal temperature reached 71°C. The cooked chops were allowed to cool for 3 min then trimmed of all outside edges and fat. Chops were cut into 1.3 cm cubes avoiding connective tissue and placed into 250 mL glass jars pre-warmed at 68°C. The samples were served to the panellist under 180-lux light in well ventilated partitioned booths. Panellist cleaned their palates between each sample with unsalted crackers and filtered water.

The panellist were asked to rate the samples on 9-point descriptive scale for initial and overall tenderness (9 = extremely tender to 1 = extremely tough), initial and sustained juiciness (9 = extremely juicy to 1 = extremely dry), and salt intensity (1 = no salt to 10 = extremely salty). Flavour desirability and overall palatability were rated on a 9-point hedonic scale (1 = not desirable to 9 = extremely desirable). Off flavour intensity was rated on a 9-point scale (9 = extremely intense to 1 = bland) and if off flavours were present, the panellist were asked to identify the most predominant descriptive classification for 'off odours’ (9 = other, 8 = unidentified, 7 = fishy, 6 = rancid/painty, 5 = stale/cardboard, 4 = piggy/barn like, 3 = metallic, 2 = off/sour, 1 = none).

### Statistical analysis

For all meat treatment group variables, least square means were generated and were tested for significance (*P* < 0.05) within GLM and ANOVA. The lipid profiles were analyzed using the MIXED procedure and significance was determined using the DIFF option and Duncan’s test to identify differences between the groups means, CON, SF, and DHA and by raw and cooked treatment effect [28]. The statistical model included the treatment effect at 1d or 3d interaction. An ordinate scale was used for the panellist evaluations of the sensory measures using Friedman test and the nominal scale was used for the biochemical measurement values, using Tukey’s HSD test.

## Results and discussion

### Pork fatty acid content

The injected loin treatments were primarily performed to determine if the DHA oil could be added at a concentration of 1 mg/g of fresh pork, without adversely affecting aroma or taste. Regular pork loins from pigs fed a standard finisher diet of, corn, barley, peas, and canola, would have ~0.5 mg of omega-3 FAME/g of meat and only ~0.02 mg of DHA FAME/g of meat [1]. Injection of the 3.1% DHA brine mixture at 10 mL/100 g into the boneless meat, increased the DHA (C22:6n-3) content 50-fold, to an estimated concentration of 1.05 mg/g of pork. This changed the fatty acid profile of omega-6: omega-3 from, 5 to 1 in the CON pork, to a ratio of 1.7 to 1 in DHA pork. The actual concentration in the loin chops was 1.16 mg/ g of raw pork (Table 2). In a previous study, 1.22 mg DHA/g of raw bacon was achieved after feeding pigs, a diet containing 0.11% DHA for 25 d –the equivalent of approximately 825 g DHA [29]. This trial achieved the 1.16 mg DHA/g level in a 10 kg loin, by injecting approximately 3.1% DHA, equivalent to approximately 32 g DHA/10 kg loin. The retention of DHA was higher after cooking at 1.46 mg/g of cooked pork. This increase was probably due to water loss evaporation by grill cooking (Table 2). The average cooking loss for all three treatments was 21.5 ± 3.04%. Conservatively, this would adjust the estimated level of DHA to approximately 0.82 mg/g of pork, if the oil was retained evenly but usually, free fatty acid content is increased by cooking [17]. The amount of 18:1cis9 and 18:2n-6 was also significantly increased in the SF and DHA treatments (Table 1) but the final concentration of 18:1cis9 and 18:2n-6 was increased less than 2-fold in the actual raw and cooked pork (Table 2).

### Colour measurements

Soy lecithin was added to the mixture because it was needed to assist the emulsion of the sunflower oil and DHA oil. The SF and DHA oil mixtures would separate into their respective phases, if left undisturbed. In the CON mixture, the addition of the soy lecithin will impart a slightly nutty aroma [30]. The vitamin E (α-tocopherol) was added to help maintain oxidative stability of the oil injection mixture and was considered as odourless and remained odourless after 6 d, as judged while training the sensory panel. The addition of vitamin E to the injected chops was expected to help prevent rancid odours and flavours [31]. The brine mixture contained 0.01% α- tocopherol, which would inject approximately 0.001% into the loins. The brine’s main ingredients were 4.8% sodium tripolyphosphate and 4.8% sodium chloride and were also determined to be odourless by the sensory panel (data not shown). Injection of a brine mixture increases tenderness and juiciness and might add some saltiness to the flavour while reducing the intensity of the pork flavour [32]. There were no difference in the behaviour of the SF or DHA oil emulsions, both oil preparations began to separate into the aqueous and oil phases in approximately 4 h after mixing and therefore needed constant stirring at 200 rpm prior to injection. The injection mixture was prepared for the experiment within 2 h before use. The colour of the oil preparations were also similar and were lightly brown, caused by the soy lecithin. There was no difference in the subjective colour of the injected loin between the CON group containing no oil and treatment groups containing SF or DHA oils, as assessed by the panellists. No difference was detected in the 3 treatments when chops were pooled according to the 12 individual animals. The panellists also did not detect any difference in the retail display, marbling, or striping between the treatment groups (Table 3). However, in the injected chops in the display case for 3 d, the panellists did give poorer scores for overall retail display and detected some color striping in the CON and treated chops. The objective colour score measured by the Minolta color meter showed a difference between the 1d and 3d chops. All the treatment groups, consistently gave higher a* index (redness), b* index (yellowness), and Chroma index (C = √a^2^ + b^2^) or 'vividness’ as the chops aged but the effect was probably not due to the DHA oil, since the CON sample showed a higher change.

**Table 3:** **Panellist assessment of *raw *** **loin chops for retail display, visual marbling, color, striping, and odours between injection treatments**

**Day 1**	**Retail display**	**Marbling**	**Color**	**Injection stripes**	**Visual discoloration**	**Off odours**	**Odour unacceptability**
CON	3.93^a^	2.38	3.91	3.09^a^	1.01	1.23	2.13^a^
SF	4.02^a^	2.91	3.45	3.25^a^	1.02	1.13	1.48^a^
DHA	3.80^a^	2.43	3.57	3.05^a^	1.02	1.18	1.80^a^
*SEM*	*0.834*	*0.049*	*0.162*	*0.867*	*0.909*	*0.364*	*0.404*
**Day 3**							
CON	2.57^b^	2.55	3.80	4.54^b^	1.07	1.25	2.09^a^
SF	2.57^b^	3.02	3.52	4.28^b^	1.04	1.34	2.46^b^
DHA	2.48^b^	2.69	3.52	4.23^b^	1.21	1.23	2.30^b^
*SEM*	*0.926*	*0.149*	*0.311*	*0.676*	*0.287*	*0.359*	*0.677*

^abc^Means within column with unique superscript differ significantly (*P* < 0.05). *SEM*; standard error of the mean.

Retail display (8 = extremely desirable to 1 = extremely undesirable).

Marbling (6 = abundant to 1 = devoid).

Color (6 = dark purple to 1 = white).

Injection stripes (7 = 100% to 1 = none).

Visual discoloration (7 = brown to 1 = none).

Off odours (4 = prevalent to 1 = none).

Odour acceptability (5 = unacceptable to 1 = acceptable).

### Measurement of oxidation

The degree of oxidation, as indicated by the amount of malonaldehyde generated by lipid peroxidation, was measured using the TBARS assay (Table 4). The injected loin chops and the purge juice from the meat samples were collected from the 1d and 3d retail display packages. On day 1, the degree of oxidation was negligible according to the assay. On day 3, the amount of oxidation in the purge but not the meat, was significantly higher compared to 1d but not between treatment groups. Meat purge represents the free flowing juices around the meat and may have a greater chance of interacting with the atmospheric oxidation. Oxidation of meat is typically recognized as an odour or colour change, characterized as 'off odours’ or a 'greying of colour’ as indicated by a reduction in the Chroma. DHA injected loins had a low increase in TBARS assay values and this corresponded to a low change in colour and off odours scores according to the consumer panel (Table 4).

**Table 4:** **The effect of injection treatments on *raw *loin chops (*n*** = 24) for TBARS estimated oxidation and Colour meter measurements L*, a*, b*, Chroma

**Day 1**	**Oxidation in purge(TBARS)**^**1**^	**Oxidation in meat (TBARS)**^**1**^	L* (lightness)	a* (redness)	b* (yellowness)	**Chroma**
CON	0.01^a^	0.011	47.47	3.67^a^	7.25^a^	8.19^a^
SF	0.01^a^	0.010	49.83	3.76^a^	8.17^a^	9.05^a^
DHA	0.01^a^	0.009	48.25	3.48^a^	7.38^a^	8.20^a^
*SEM*	*0.178*	*0.501*	*0.137*	*0.862*	*0.126*	*0.254*
**Day 3**						
CON	2.37^b^	0.015	48.76	4.17^b^	9.09^b^	10.03^b^
SF	2.66^b^	0.018	50.25	4.26^b^	9.91^bc^	10.83^b^
DHA	2.21^b^	0.015	49.57	3.75^a^	9.11^b^	9.87^b^
*SEM*	*0.189*	*0.122*	*0.445*	*0.509*	*0.098*	*0.161*

Means with column with unique superscript differ significantly (*P* < 0.05). *SEM;* standard error of mean.

^1^TBARS; Thiobarbituric Acid Reactive Substances (mg malonaldehyde/kg of meat).

L*:0 = black, 100 = white; a*: red(+) to green(-), b*: yellow(+) to blue(-).

The DHA oil had over twice the estimated iodine index value at 271.36 than the sunflower oil at 110.43 (Table 1) and therefore the potential for lipid oxidation would be expected to be greater [33]. The sensory panellists judged the DHA and SF injected raw pork to be both worse for odour unacceptability after 3 d, than the CON pork (Table 3). The SF treatment actually had a higher estimated iodine value in both raw and cooked pork (Table 2), compared to the DHA treatment. This higher oxidation was indicated by a non-significant higher 3d TBARS values for SF at 2.66 compared to 2.21 for DHA in the purge juice (Table 4) and also by the panellist assessment of 'off flavours’ in the cooked pork (Table 5) but the CON cooked pork also rated high, which indicates that there is more to off flavours perception than just TBARS values.

**Table 5:** **Panellist assessment on *cooked *** **loin chops for, tenderness, juiciness, saltiness, and overall palatability between injection treatments**

	**Cook time (min.)**	**Initial tenderness**	**Initial juiciness**	**Salt intensity**	**Flavour desirability**	**Pork flavour**	**Off-flavours**	**Sustained juiciness**	**Overall tenderness**	**Overall palatability**
CON	18.25^a^	6.91	5.32^a^	5.04^a^	4.71	4.46	1.14^a^	5.77	7.25	4.18
SF	14.87^b^	6.73	5.77^ab^	5.22^a^	4.75	4.43	1.16^a^	6.07	7.31	4.27
DHA	15.25^b^	7.11	6.39^b^	4.61^b^	4.62	4.20	0.75^b^	6.23	7.45	4.25
*SEM*	*0.039*	*0.037*	*0.025*	*0.096*	*0.832*	*0.595*	*0.052*	*0.265*	*0.175*	*0.926*

^ab^Means with column with unique superscript differ significantly (*P* < 0.05). *SEM* ; standard error of mean.

Tenderness (9 = extremely tender to 1 = extremely tough); juiciness (9 = extremely juicy to 1 extremely dry); flavour, pork flavour, palatability and sustained juiciness, overall tenderness and overall palatability (9 = extremely desirable to 1 = not desirable).

Off flavour and salt intensity (9 = extremely intense to 1 = none).

### Sensory taste panel

The odour and sensory evaluations were made with a eight member trained taste panel. Before the trial, the panellist were asked to evaluate the chemicals: 1-hexanal, butanoic acid, docosahexanoic acid (DHA) oil, sunflower oil (SF) and DHA or SF oil plus soy lecithin. The chemicals 1- hexanal and butanoic acid were chosen as probable breakdown products of oxidation of DHA, caused by air exposure which leads to oxygen cleavage at double bonds [34,35]. The 1-hexanal was described as 'stale’ and 'grassy’ and the butanoic acid was describes as 'rancid butter’. The DHA and DHA plus lecithin was initially odourless and nutty but was later described as fishy after exposure to air for >1 h. The SF and SF + lecithin was odourless then described as 'oily’ or 'stale’ after being exposed to air for more than 1 h. The raw chops were allowed to reach room temperature after 1 h before the chops were evaluated for odours. On day 1 and 3, the vitafilm wrapped, raw loin chops were assessed for odours. The raw chops were rated as generally acceptable for overall odours on day 1 and day 3 (Table 3). There was a noticeable drop in the 'unacceptable odours’ score by day 3 but this was still within the partially acceptable to neutral range and consistent between all treatments. There was no difference between the treatments and the scores were very low and unchanged for 'off’ odours in the 1 d and 3 d chops.

Chops were cooked, 24 h after injection, and were offered to the panellists which evaluated them for palatability and sensory flavours. The amount of cooking loss (%) was not significantly different between the CON (22.2 ±2.8%), the SF (19.9 ±3.6%) or the DHA (22.4 ±2.2%) treatments. There was very little difference between the treatments, according to the taste panellist as well (Table 5). The injected cooked chops rated highly for scores of, initial and sustained juiciness, initial and overall tenderness, and salt intensity. Initial juiciness was scored the highest in the DHA injected chops (Table 5). Salt intensity score was reduced by the addition of DHA and should be investigated further. The addition of tripolyphosphate and water to the chops has been used in the pork industry for over a decade and in the poultry industry since 1954 [12]. It has been proposed that polyphosphate has two effects of depolymerisation of myosin filaments and weakening the binding of myosin with actin, thus promoting muscle fibre relaxing [32]. This also would permit polyphosphate treated meat to retain more water. The panellists did not score any differences in the flavour of the 1d cooked chops between the treatment groups. The flavours were scored as bland, regardless of the treatments, and the overall palatability and pork flavour scored as 'slightly desirable’ to 'neutral’ in the trial. This is in agreement with previous sensory studies [32] which noted that the brine injected meat has only a minimal increase on saltiness scores and a less intense, pork flavour. It has been speculated that the flavour of brine injection, dilutes the carbohydrates, proteins and lipids and washes away the Maillard reactions complexes, which give meat its’ roasted flavour [36]. If a panellist did mark the injected chops for 'off flavours’, they scored the sample as very low and gave a description as 'stale’ or 'piggy’ and surprisingly, the off flavours score were higher in the untreated CON and SF injected cooked pork than the DHA injected cooked pork (Table 5). It has been noted that DHA triacylglycerol can impart umami and flavour and supress bitterness in certain taste panels [37].

## Conclusions

The injection of pork loins with 3.1% DHA in a tripolyphosphate brine mixture was rated to be 'desirable’, by trained taste panellist. The trained taste panel also scored the cooked DHA injected pork better at surviving against 'off’ flavours, than CON and SF injected pork. Increasing the lipid content ~ 0.3% by weight in the loins may have improved the juiciness of the cooked loin, especially since the IMF of pork was >2%. DHA content was improved approximately 50-fold to 1.16 mg DHA/g of raw pork, which converts to 116 mg of DHA in a typical 100 g serving of pork. The DHA content was further improved by cooking on a grill to 146 mg of DHA/100 g of pork. This would meet over half the adult human recommended daily requirements for DHA omega-3 fatty acid [3]. The injection of DHA oil added to the nutritional value of the pork and will help in reducing plasma triglycerides of consumers [38].

## Competing interests

The authors declare that they have no competing interest.

## Authors’ contributions

WJM designed and completed the overall design and statistical analysis of the experiment and was the main author of the manuscript. LG directed the sensory panel and assessment. JA performed the biochemical measurement and BU was responsible for the injection and preparation of marinades. MD and TT performed the fatty acid analysis. All authors read and approved the final manuscript.
